# Cystatin C alleviates unconjugated bilirubin-induced neurotoxicity by promoting bilirubin clearance from neurocytes via exosomes, dependent on hepatocyte UGT1A1 activity

**DOI:** 10.1515/tnsci-2022-0357

**Published:** 2024-10-14

**Authors:** Yating Du, Zhenkun Li

**Affiliations:** Department of Anesthesiology, Beijing Friendship Hospital, Capital Medical University, No. 95 Yong-An Road, Xi-Cheng District, Beijing, 100050, People’s Republic of China; Beijing Clinical Research Institute, Beijing Friendship Hospital, Capital Medical University, No. 95 Yong-An Road, Xi-Cheng District, Beijing, 100050, People’s Republic of China

**Keywords:** cystatin C, unconjugated bilirubin, exosome, UDP-glucuronyl transferase1A1

## Abstract

There is an urgent need to identify effective drugs for the treatment of nerve injury caused by unconjugated bilirubin (UCB). Our previous research found that cystatin C (CST3) alleviates UCB-induced neurotoxicity by promoting autophagy in nerve cells, but that autophagy inhibitors did not completely inhibit the effects of CST3. This study investigated whether CST3 could alleviate the neurotoxicity of UCB by promoting the secretion and transport of exosomes containing UCB to the liver for metabolism. It demonstrated that hyperbilirubinemia mice treated with CST3 had a higher number of serum exosomes than those in hyperbilirubinemia mice treated with phosphate-buffered saline. CST3-mediated protection against UCB-induced damage was abolished when autophagy and extracellular vesicle inhibitors were used in combination. The number of exosomes in the CST3 overexpression group was higher than that in the control group. Molecular docking experiments showed that UCB and CST3 had high docking score (−8.2). These results suggest that UCB may be excreted from cells by exosomes, and CST3 may promote this process by binding to UCB and entering the exosomes. We demonstrated that the effect of CST3 relied on liver cells with normal UDP-glucuronyl transferase1A1 (UGT1A1) activity in a coculture system of HT22 and L02 cells. CST3 levels were lower in exosomes secreted by L02 cells than in those secreted by human umbilical vein endothelial cells (HUVECs), whereas CST3 levels were higher in the culture supernatants of L02 cells than in the culture supernatants of HUVECs. This suggests that UCB exosomes in L02 cells may be released and internalized by CST3 and that UCB is then processed by UGT1A1 to conjugate UCB, thus reducing its toxicity. These results suggest that CST3 might alleviate UCB-induced neurotoxicity by promoting the clearance of UCB from cells via exosomes and that these effects are dependent on UGT1A1 activity in liver cells.

## 1 Introduction

Hyperbilirubinemia can cause damage to the nervous system [1]. Given that unconjugated bilirubin (UCB) possesses the ability to traverse the blood–brain barrier (BBB), its accumulation within the body has the potential to cause significant brain damage, a condition commonly referred to as bilirubin encephalopathy [2]. Severe hyperbilirubinemia can cause kernicterus in neonates and children [3] and the injury may be irreversible [4]. Therefore, close attention should be paid to the neurological status of patients with hyperbilirubinemia and timely and effective treatment measures should be taken to reduce the damage.

Hyperbilirubinemia can be treated with blue light therapy and plasma exchange, if necessary. Although plasma exchange can rapidly reduce the blood UCB content, it has serious complications [5]. Phototherapy can cause permanent hyperkeratosis and rash, which seriously affects patients’ quality of life. Moreover, the effectiveness of phototherapy decreases due to skin thickening decreasing the clearance of UCB isomers by the hepatic and biliary systems in adults [6]. Therefore, drugs that are effective at treating UCB nerve injury are urgently needed.

Cystatin C (CST3) is a low molecular weight protein produced by nucleated cells [7], and is the most important extracellular inhibitor of cysteine proteinases [8]. It has been reported that CST3 could be released by exosomes [9,10]. Besides, CST3 enhances secretion of extracellular vesicles (EVs) in primary cortical neurons and primary cortical smooth muscle cells [11]. Our previous research found that CST3 alleviates UCB-induced neurotoxicity by promoting autophagy in nerve cells and that autophagy inhibitors can significantly but not completely inhibit the effect of CST3 [12]. We speculate that CST3 could alleviate UCB-induced neurotoxicity through other pathways. The liver is the most important organ for UCB metabolism [13], and there is a close relationship between the liver and the brain. The liver and brain can interact in a bidirectional manner via autonomic nervous system innervation [14]. Cognitive impairment is prevalent in patients with primary biliary cirrhosis [15]. In patients with liver failure, hepatic encephalopathy manifests as abnormal behavior and impaired cognition [16].

Inflammatory liver injury is associated with changes in cerebral neurotransmission that results in sickness behaviors [17]. Brain Aβ deposits are reported to originate in the liver and the liver is involved in the clearance of circulating Aβ in the peripheral blood [18,19]. A better understanding of the brain–liver axis might help to explain the abnormal cognitive and behavioral phenomena observed in liver diseases [20].

Exosomes are EVs secreted by cells, with a double-layered membranous structure, and sizes ranging from 30 to 200 nm [21]. Exosomes have formed a novel intercellular communication system [22,23]. It has been reported that CST3 is also secreted by mouse primary neurons in association with exosomes [24]. In addition, CST3 enhances EV release, which can counter the deleterious effects of endosomal–lysosomal system dysfunction in neurodegenerative disorders [11]. However, the relationship between CST3 and the exosomes in UCB-treated HT22 cells remains unclear. We speculate that CST3 could alleviate the neurotoxicity of UCB by promoting the secretion and transport of exosomes containing UCB to the liver for metabolism.

## 2 Materials and methods

### 2.1 Isolation of exosomes

Exosomes were isolated using the salting-out method (serum, Invitrogen™) and ultracentrifugation (cell culture medium). The serum sample was centrifuged at 2,000 × *g* for 20 min. The supernatant was centrifuged again at 10,000 × *g* for 20 min. Phosphate-buffered saline (PBS) of 0.5 volume and 0.3 volume exosome isolation reagent were added to the clarified serum. The sample was centrifuged at 10,000 × *g* for 5 min and the supernatant was aspirated and discarded. Finally, the tube was centrifuged for 30 s at 10,000 × *g* and the residual supernatant was discarded by careful aspiration. The cell culture medium was centrifuged at 300 × *g* for 10 min, then at 2,000 × *g* for 10 min, 10,000 × *g* for 30 min, and 140,000 × *g* for 90 min. Exosomes were identified using transmission electron microscopy (uranyl acetate). Criteria/standard: double membrane structure, particle size 30–200 nm.

### 2.2 Exosome quantification

The exosome quantification was performed by exosome quantitative kit (abs50054; Absin) following the manufacturer’s instructions.

### 2.3 Molecular docking

We analyzed the protein structures of UBC and CST3 using molecular docking by 1-click-docking (https://mcule.com/apps/1-click-docking/). Bilirubin Isomeric SMILES: CC1═C(NC(═C1CCC(═O)O)CC2═C(C(═C(N2)/C═C\3/C(═C(C(═O)N3)C)C═C)C)CCC(═O)O)/C═C\4/C(═C(C(═O)N4)C═C)C; cystatin C ID: 1g96.

### 2.4 Cell culture

HT22 cells, L02 cells, and human umbilical vein endothelial cells (HUVECs) were cultured in RPMI-1640 medium (Sigma-Aldrich, St Louis, MO, USA) supplied with 10% FBS. The medium was changed every 2–3 days. CST3 proteins were obtained from ProSpec (Rehovot, Israel). The proteins were dissolved in double-distilled water. The cell confluency when the cells were treated with CST3 was approximately 40%. About 100 µg/mL of UCB was added to the cells to establish a high UCB cell model.

### 2.5 Co-culture experiment

Co-culture experiment was performed using 24-well transwell with 0.4 µm pore size (Costar). About 1,000 cells were added into the upper chamber and 2,000 cells were added into the lower chamber. These cells were co-cultured for 12 h and then different treatments were performed [25].

### 2.6 Establish of UGT1A1-KO L02 cells

We used the pSpCas9(BB)-2A-GFP (PX458) (LM-2258, LMAI Bio) to establish UGT1A1-KO L02 cells. The gRNA was designed by AGTC knockout guide designer. The gRNA sequence was F-CACCGTACCCTGTGCCATTCCAAAGGG, R-CATGGGACACGGTAAGGTTTCCC. Lipofectamine™ 3000 (L3000015, Invitrogen™) was used for transfection. The effects were verified by quantitative polymerase chain reaction (qPCR) and western blotting.

### 2.7 qPCR

Total RNA was extracted from HT22 cells using TRIzol reagent (Tiangen, Beijing, China). We synthesized cDNA using the FastQuant RT Kit (Tiangen) following the manufacturer’s instructions. qPCR was performed on an IQ5 thermal cycler (Bio-Rad, Hercules, CA, USA) using the following cycling conditions: predenaturation at 95°C for 15 min, 40 cycles of denaturation at 95°C for 10 s, annealing at 60°C for 15 s, extension at 72°C for 20 s, and 71 cycles of melt curve analysis at 60°C for 10 s. The following primers were used: forward UGT1A1 primer sequence, ACTGTTGATCCCAGTGGATGG; reverse UGT1A1 primer sequence, TCTGATGTACAACGAGGCGT; forward β-actin primer sequence, CCTCGCCTTTGCCGATCC; and reverse β-actin primer sequence, CGCGGCGATATCATCATCCAT.

### 2.8 Western blotting

Proteins were extracted from brain samples using a Protein Extraction Kit (JiangSu CoWin Biotech Co., Ltd, Jiangsu, China) and quantified using BCA reagents (JiangSu CoWin Biotech). Proteins were separated using sodium dodecyl-sulfate polyacrylamide gel electrophoresis at 160 V on a 12% gel (JiangSu CoWin Biotech) for 1 h and then transferred to a 0.22 μm nitrocellulose filter membrane at 200 mA for 3 h. The membranes were incubated with autophagy-related primary antibodies diluted as follows: anti-UGT1A1 antibody (ab170858; Abcam, Cambridge, UK) (1:1,000) and anti-GAPDH antibody (ab181602;, Abcam) diluted to 1:2,000. The membranes were then incubated with secondary antibodies (abs20001 and abs20002; Absin) diluted to 1:5,000. The membranes were washed, and bands were visualized using enhanced chemiluminescence immunoblotting detection reagents (Thermo Fisher Scientific, Waltham, MA, USA). For autophagic proteins, bafilomycin A1 was used at a final concentration of 200 nM and added during the last 3 h. Images were obtained using Image Lab Software (Bio-Rad, Hercules, CA, USA), and semi-quantitative analysis was performed by normalizing the protein bands to the internal control GAPDH.

### 2.9 Cytotoxicity assay

We performed MTT assays (Solarbio, Beijing, China) to examine the viability of HT22 cells. Cells treated with solvent were used as the negative control. HT22 cells were plated at a density of 2,000 cells/well and allowed to adhere for 6 h, followed by treatment with CST3 protein and UCB. Wells containing 100 μL medium alone (without cells) were used as blank controls. MTT assays were performed after treatment for 48 h. The blank control was served as the baseline. Each experiment was repeated three times, and the results were presented as the percentage of viable cells calculated using the following equation:

\[({\mathrm{Mean\; absorbance\; of\; experimental\; well}}\left/{\mathrm{mean\; absorbance\; of\; control\; well}})\times 100={\mathrm{relative\; cell\; viability\; to\; control}}]\]

### 2.10 Enzyme-linked immunosorbent assay (ELISA)

The concentration of CST3 was determined using a CST3 ELISA kit (Cyagen, Santa Clara, CA, USA) according to the manufacturer’s instructions.

### 2.11 Statistical analyses

Statistical analyses were performed using SPSS version 22.0 (IBM Corp., Armonk, NY, USA). After the normality and variance homogeneity tests of the measured data, comparisons between different groups were performed using Student’s *t*-test or one-way analysis of variance. Data were shown as mean ± SD. *p*-Values ≤0.05 was considered statistically significant.

## 3 Results

### 3.1 CST3 is associated with exosomes secretion

**Figure 1:**
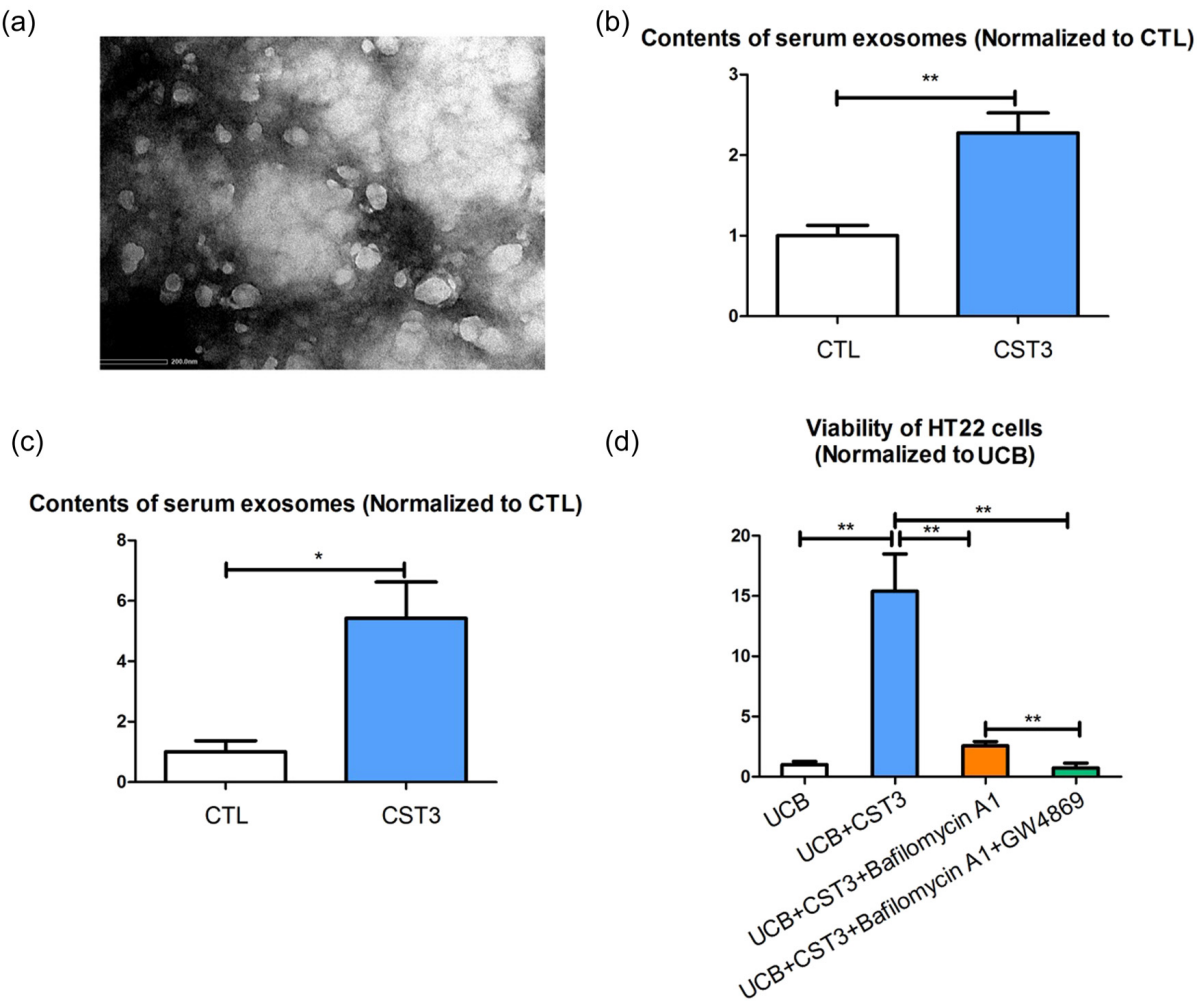
CST3 is associated with exosomes secretion. (a) The extracted exosomes were identified by electron microscopy. (b) The serum exosomes in hyperbilirubinemia mice treated with CST3 and hyperbilirubinemia mice treated with PBS. (c) The exosomes content in the culture supernatant of CST3 overexpressing HT22 cells and the control HT22 cell culture supernatant. (d) The effect of exosomes on CST3 alleviating UCB-induced damage. *p ≤ 0.05; **p ≤ 0.01.

The extracted exosomes were identified by electron microscopy (Figure 1a). The number of serum exosomes was higher in hyperbilirubinemia mice [12] treated with CST3 than in those treated with PBS (Figure 1b). We speculated that CST3 promoted exosome secretion. We established overexpressing CST3 HT22 cells [12] and measured the number of exosomes in the cell culture supernatant. The number of exosomes in the culture supernatant of CST3 overexpressing HT22 cells was higher than that in the control HT22 cell culture supernatant (Figure 1c). In addition, the effect of CST3 on alleviating UCB-induced damage was inhibited by EV inhibitors, and CST3-mediated protection against UCB-induced damage was completely abolished when autophagy and EV inhibitors (bafilomycin A1 and GW4869) were used in combination (Figure 1d).

### 3.2 CST3 promotes UCB excretion from cells in exosomes

**Figure 2:**
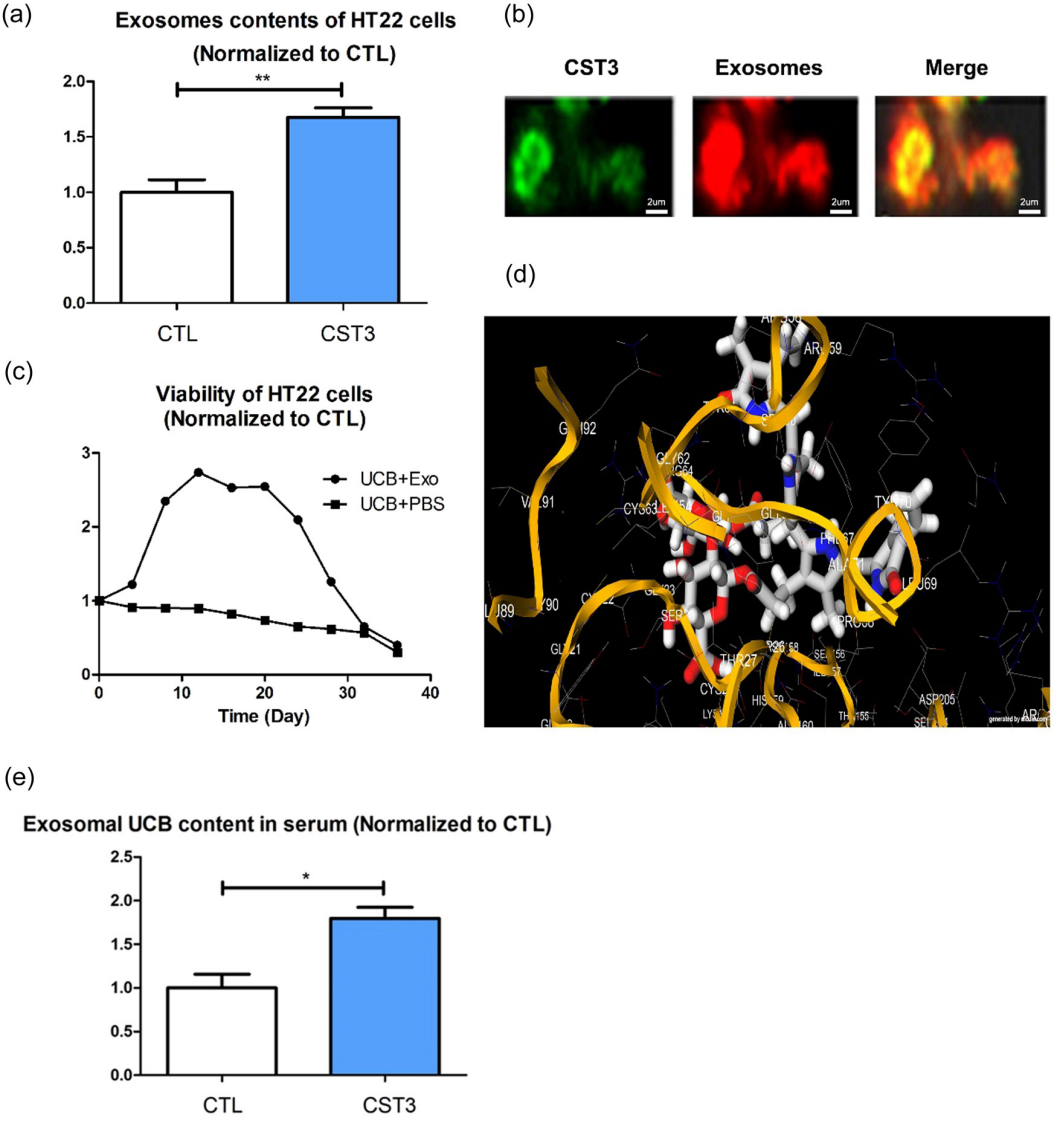
CST3 may promote UCB excreted out of cells in the form of entering into exosomes: (a) exosomes content in the CST3 overexpression group and the control group, (b) DiL staining of exosomes, (c) different time points of cell viability in the exosomes group and control group, (d) molecular docking between UCB and CST3, and (e) serum exosomal bilirubin levels. *p ≤ 0.05; **p ≤ 0.01.

We speculated that the mechanism for alleviating UCB-induced damage by promoting exosome secretion was the clearance of UCB by promoting UCB bound to CST3 into exosomes and excretion from the cell. However, this effect decreased over time due to exosome rupture or internalization. We found that the number of exosomes in the CST3 overexpression group were higher than that in the control group (Figure 2a). DiL staining confirmed that CST3 had entered the exosomes (Figure 2b). We extended and increased the time points for measuring cell viability and found that the viability of cells in the exosome and control group (PBS) tended to be consistent over time (Figure 2c). In addition, molecular docking experiments revealed that UCB and CST3 had a high docking score (−8.2; Figure 2d). Besides, we tested the bilirubin levels in exosomes. Serum exosomal bilirubin levels significantly increase in CST3-treated group than that in CTL group (Figure 2e). These results suggest that UCB may be excreted from cells by exosomes, and that CST3 may promote this process by binding to UCB and entering the exosomes.

### 3.3 UCB exosomes entered liver cells

**Figure 3:**
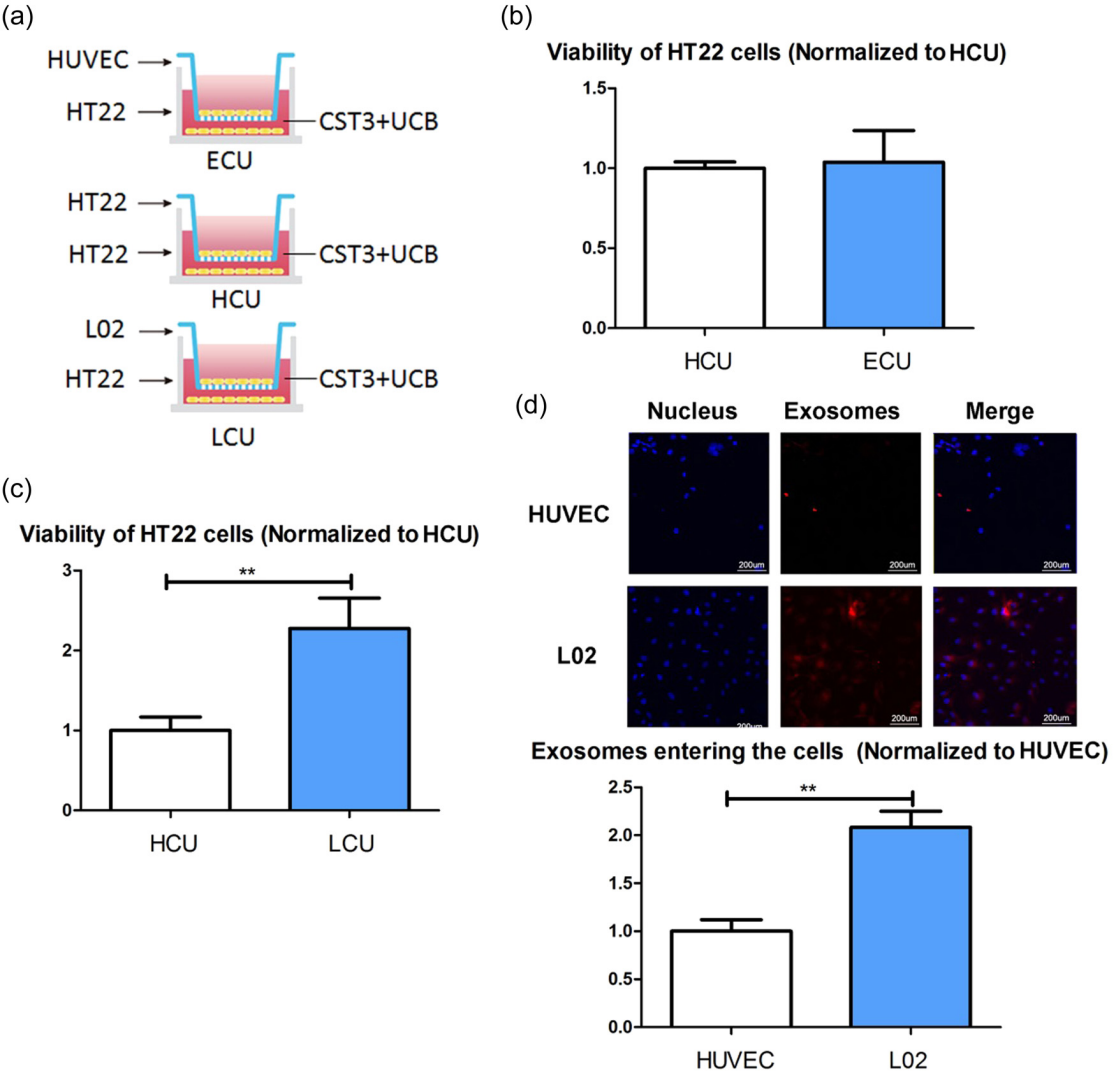
UCB exosomes entered liver cells: (a) establish co-cultured systems, (b) viability of the ECU HT22 cells and HCU HT22 cells, (c) viability of the LCU HT22 cells and the HCU HT22 cells, and (d) exosomes uptake ability of L02 cells and HUVECs. ECU: co-cultured with HUVECs and treated with CST3 + UCB; HCU: co-cultured with HT22 and treated with CST3 + UCB; LCU: co-cultured with L02 cells and treated with CST3 + UCB. **p ≤ 0.01.

To explore the possible destination of UCB exosomes *in vivo*, the co-cultured systems were established (Figure 3a). We co-cultured HT22 cells with HUVECs and hepatocytes (L02 cells), respectively. There was no difference in viability between the HT22 cells co-cultured with HUVECs and treated with CST3 + UCB (ECU cells) and HT22 cells co-cultured with HT22 cells and treated with CST3 + UCB (HCU cells) (Figure 3b). However, compared with the HCU cells, the viability of the HT22 cells co-cultured with L02 cells and treated with CST3 + UCB (LCU cells) showed increased viability (Figure 3c). To verify the exosome uptake ability of L02 cells and HUVECs, we stained the exosomes and measured the amount of exosomes entering the cells. The results showed that more exosomes entered L02 cells than into HUVECs (Figure 3d).

### 3.4 Function of CST3 via exosomes is dependent on UDP-glucuronyl transferase1A1 (UGT1A1) in liver cells

**Figure 4:**
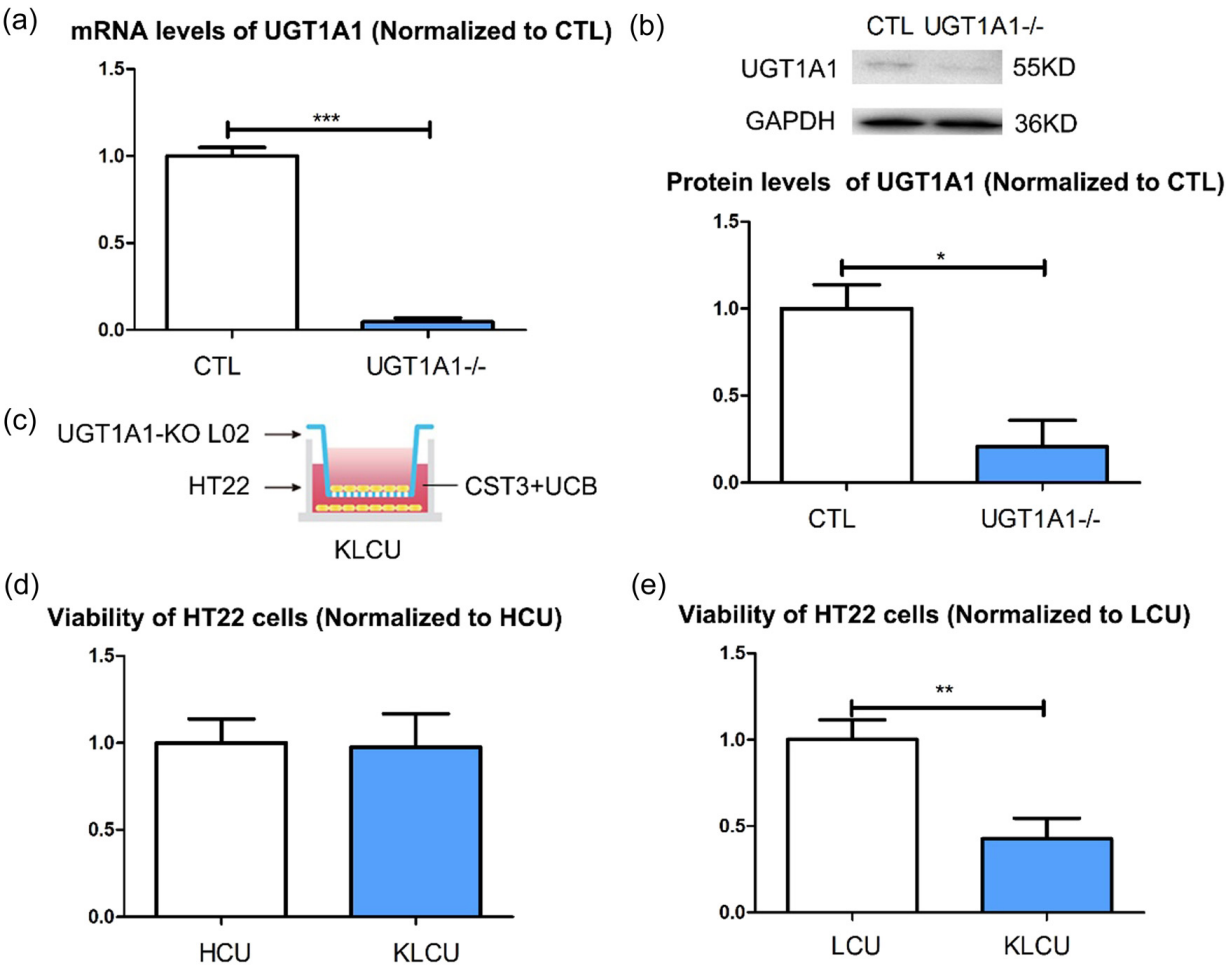
Function of CST3 via exosomes is dependent on liver cells UGT1A1s: (a) verification of UGT1A1 knockout L02 cells in mRNA level, (b) verification of UGT1A1 knockout L02 cells in protein level, (c) establish KLCU co-cultured system, (d) viability of the KLCU HT22 cells and HCU HT22, and (e) viability of the KLCU HT22 cells and LCU HT22. KLCU: co-cultured with UGT1A1-KO L02 cells and treated with CST3 + UCB. *p ≤ 0.05; **p ≤ 0.01; ***p ≤ 0.001.

The liver is a major organ for UCB metabolism. Co-culturing of UCB-treated HT22 cells with L02 cells significantly improved their viability, indicating that liver cells are the recipient cells in this process. UGT1A1 plays an important role in UCB metabolism in liver cells. To test this hypothesis, we generated UGT1A1 knockout L02 cells (Figure 4a and b). There was no significant difference in viability between the HT22 cells co-cultured with UGT1A1-KO L02 cells and treated with CST3 + UCB (KLCU cells) (Figure 4c) and the HCU cells (Figure 4d), whereas the viability of KLCU cells was significantly lower than that of LCU cells (Figure 4e).

### 3.5 UCB exosomes were internalized by liver cells

**Figure 5:**
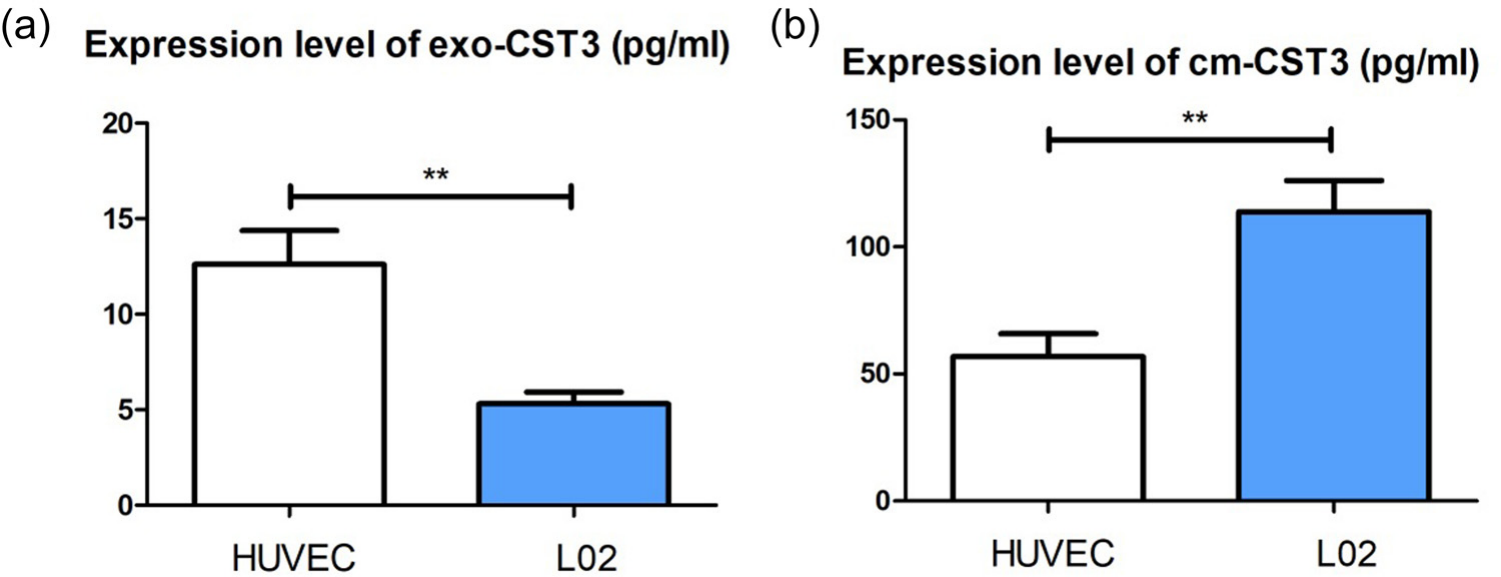
UCB exosomes were internalized by liver cells: (a) CST3 levels in exosomes secreted from L02 cells and HUVECs by ELISA and (b) CST3 levels in the culture supernatant of L02 cells and HUVECs by ELISA. **p ≤ 0.01.

To explore whether HUVECs and L02 cells differ in their handling of internalized UCB exosomes, we collected cell culture supernatants after treating HUVECs and L02 cells with UCB exosomes for 48 h and measured the number of exosomes and the CST3 levels (subtracting the background value). The CST3 levels in exosomes secreted by L02 cells were lower than in those secreted by HUVECs (Figure 5a), whereas the CST3 levels were higher in the culture supernatant of L02 cells than in that of HUVECs (Figure 5b). This suggested that UCB exosomes in L02 cells might be released and internalized by CST3, and that UCB was then processed by UGT1A1 to conjugated UCB thus reducing UCB toxicity.

## 4 Discussion

Multiple studies have demonstrated that CST3 plays a protective role in the brain [26,27], possibly through pathways associated with autophagy or inhibition of cysteine proteases [28,29]; however, the relationship between CST3 and hyperbilirubinemia, and the function of exosomes in hyperbilirubinemia is unclear. Exosomes are EVs ranging from 30 to 200 nm in size and are secreted by cells with various functions [22,23]. CST3 is also secreted by mouse primary neurons in association with exosomes [24], and CST3 enhanced EV release, which could counter the deleterious effects of endosomal–lysosomal system dysfunction in neurodegenerative disorders [11]. However, the relationship between CST3 and the exosomes in UCB-treated HT22 cells is unclear. This study showed that CST3 can enhance the secretion of exosomes in HT22 cells. The liver is a major organ for UCB metabolism. However, the relationship between the liver and brain in bilirubin encephalopathy has been seldom studied. Our data showed that this increases the viability of bilirubin-treated HT22 cells, in a liver UGT1A1-dependent manner. This process might be achieved by CST3 promoting bilirubin release from neurons.

Brain diseases are prevalent, because the currently available central nervous system drugs cure only an extremely small percentage of brain disease [30]. A major obstacle is that very few drugs can cross the BBB to reach their targets within the brain parenchyma [31]. Cathepsin B can cross the BBB [32], and CST3 is a small molecular protein that may penetrate BBB (Figure S1) and can easily be excreted from the body.

In conclusion, this study showed that CST3 could alleviate UCB-induced neurotoxicity by promoting exosomes secretion, and that this protection effect is dependent on UGT1A1 activity in liver cells.

## Supplementary Material

Supplementary Figure
