# Supplementary material for: Cystatin C alleviates unconjugated bilirubin-induced neurotoxicity by promoting bilirubin clearance from neurocytes via exosomes, dependent on hepatocyte UGT1A1 activity
Source: Transl Neurosci. 2024 Oct 14;15(1):20220357. doi: 10.1515/tnsci-2022-0357 (PMC11491770; doi:10.1515/tnsci-2022-0357)
Supplement: Supplementary Figure [file tnsci-2022-0357-sm.pdf]

Supplementary material

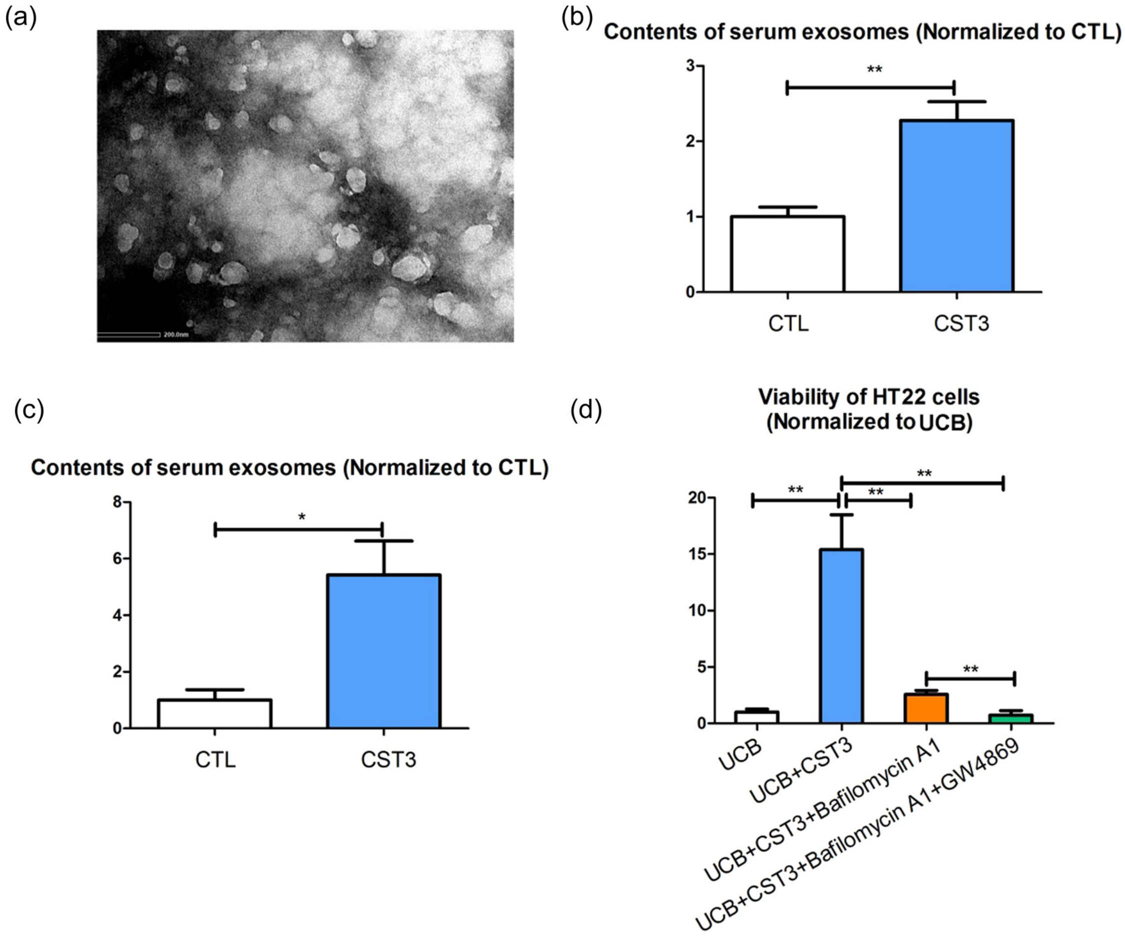

Figure S1: The content of proteins in transwell lower chamber were detected.

Open Access. © 2024 the author(s), published by De Gruyter. This work is licensed under the Creative Commons Attribution 4.0 International License.
